# Shifting focus: The impacts of sustainable seafood certification

**DOI:** 10.1371/journal.pone.0233237

**Published:** 2020-05-20

**Authors:** Ingrid van Putten, Catherine Longo, Ashleigh Arton, Matt Watson, Christopher M. Anderson, Amber Himes-Cornell, Clara Obregón, Lucy Robinson, Tatiana van Steveninck

**Affiliations:** 1 CSIRO, Oceans and Atmosphere, Hobart, Tasmania, Australia; 2 Centre for Marine Socioecology, University of Tasmania, Hobart, Tasmania, Australia; 3 Marine Stewardship Council (MSC), Snow Hill, London, United Kingdom; 4 Marine Stewardship Council (MSC), Marine Terrace, Fremantle, WA, Australia; 5 University of Washington, School of Aquatic and Fishery Sciences, Seattle, WA, United States of America; 6 Food and Agriculture Organization of the United Nations, Rome, Italy; 7 College of Science, Health, Engineering and Education, Harry Butler Institute, Centre for Sustainable Aquatic Ecosystems, Murdoch University, Murdoch, Australia; 8 CSIRO, Oceans & Atmosphere, Perth, Western Australia, Australia; 9 Oceans Graduate School, University of Western Australia, Perth, Western Australia, Australia; 10 Leiden University, Science Based Business, Leiden, The Netherlands; Universitá Cattolica del Sacro Cuore, ITALY

## Abstract

Alongside government driven management initiatives to achieve sustainable fisheries management, there remains a role for market-based mechanisms to improve fisheries outcomes. Market-based mechanisms are intended to create positive economic incentives that improve the status and management of fisheries. Research to understand consumer demand for certified fish is central but needs to be mirrored by supply side understanding including why fisheries decide to gain or retain certification and the impact of certification on them and other stakeholders involved. We apply semi-structured interviews in seven different Marine Stewardship Council (MSC) certified fisheries that operate in (or from) Western Australia with the aim of better understanding fisheries sector participation in certification schemes (the supply side) and the impacts and unintended benefits and costs of certification. We find that any positive economic impacts of certification were only realised in a limited number of MSC fisheries in Western Australia, which may be explained by the fact that only a small proportion of Western Australian state-managed fisheries are sold with the MSC label and ex-vessel or consumer market price premiums are therefore mostly not obtained. Positive impacts of certification in these Western Australian fisheries are more of a social and institutional nature, for example, greater social acceptability and increased efficiency in the governance process respectively. However, opinion is divided on whether the combined non-monetary and monetary benefits outweigh the costs.

## 1 Introduction

Globally, fisheries make an important contribution to many national economies [1]. In many low- and medium-income countries fisheries underpin food security [1, 2] and, more broadly, fisheries contribute to coastal community livelihoods. Despite the cultural, social and economic importance of fisheries [3], and even though there have been improvements globally, overfishing and unsustainable fishing still remains in some places and stocks [4]. National governments and global or regional institutions (such as the Food and Agriculture Organization of the United Nations (FAO)) have been pursuing many different management approaches [5–8], such as Ecosystem-Based Fisheries Management [9, 10] to improve fisheries outcomes. Implementation of these approaches is often complex, but further improvement of fisheries management continues to be essential [11]. Alongside government driven management initiatives, there remains a role for market-based mechanisms to further improve fisheries outcomes [12].

The option of fisheries certification as a potential market-based mechanism to improve fisheries outcomes was first raised in the 1990s [13] in response to the collapse of the cod fishery on the Grand Banks [14]. Market-based mechanisms are intended to create positive economic incentives that improve the status and management of fisheries [15]. A pathbreaker in the market-driven space, the Marine Stewardship Council (MSC) was established in 1997 jointly by the World Wildlife Fund and Unilever [15, 16]. Unilever’s intention as one of the world’s largest seafood processors at the time was to buy all their fish from certified sustainable sources by 2005 [17]. As of 2018, 361 fisheries worldwide are MSC certified and 109 are in the process of becoming certified, accounting for 15% of global wild capture production [18]. In addition, many alternative fisheries certification schemes now exist, although the MSC remains the largest in number and geographical spread [19, 20].

Obtaining MSC certification of a fishery requires meeting all MSC’s Fishery Standard requirements, as verified by an independent third party (i.e., Conformity Assessment Body (CAB)). A certificate lasts five years, with a surveillance audit undertaken each year. Certified fisheries need to undertake and pay for all assessments and surveillance audits. At the end of five years, fisheries wishing to remain certified must begin the full cycle again (i.e., get recertified) [21]. To enable a product to be sold to the public with the MSC ecolabel, each actor involved in its supply chain (i.e., processors, traders, buyers, and retailers) must hold valid MSC Chain of Custody certificates in order to assure full traceability back to the certified fishery (or fisheries) it is sourced from.

There remains much discussion in the literature about whether the environmental and sustainability goals and objectives of the many existing seafood certification schemes are being achieved [19, 22–28]. Despite the ongoing debate about the environmental outcomes, many different certification and rating schemes have entered the market [29] since the 1990s. Different certification programs take different approaches both in recruiting new fisheries into compliance with the standard, and in marketing their labels. The certification landscape has become a busy one [30], which can be confusing to potential new fishery participants and consumers of certified products [31, 32]. From a consumer perspective, studies suggest that brand recognition is not evenly distributed around the world, but in the US and Europe, the MSC is more recognized than other schemes [32–36].

Europe and North America account for 45 per cent of global certified seafood production although their contribution to global seafood production is only 15 per cent. Demand for certified fisheries products is mainly driven by manufacturers and retailers in these same developed-country markets. The demand is predominantly for high value species from high visibility fisheries with strong management capacity [37]. The demand for certified product appears adequate to support the continued existence of certification schemes, supported by reports that some retailers have been unable to meet their supply needs [38].

Understanding the demand for certified fish, including the demand by supermarkets who are important buyers of certified fish, is central to the continued existence of market-based certification schemes [39]. However, the supply side—including fishing fleets, primary processors and distributors—also needs to be understood to ensure the availability of certified fish. Understanding the supply side can give insight into why fisheries decide to gain or retain certification, including their perceptions and experiences of the impact of certification. There is some evidence, for instance, that price premiums have drawn fishers and the fisheries sector more broadly into certification [14]. Price premiums have been captured at different levels of the supply chains [40]. For instance, a price premium for MSC certified Swedish Eastern Baltic cod was achieved for retailers but not for fishers [41]. But it is increasingly debated if these price premiums currently still exist in some markets for certified fish [42–45]. Market access seems to be a more commonly realised and identifiable economic driver for fisheries to seek MSC certification. Market access becomes key to fisheries when buyers in the supply chain commit to sourcing some or all seafood from sustainable sources [42, 46–49]. However, the economic benefit of market access can vary significantly, depending on availability and abundance of competing products on the market, and environment or trade conditions.

The ability to capture benefits can also vary throughout the supply chain, with processors potentially retaining price premium benefits, while producers might only receive the benefit of being granted access to a new market [41]. Indeed, the drivers and incentives are further complicated by the multiple pathways that harvest from certified sources may take before it arrives to consumers. For some schemes, such as the MSC, exhibiting the sustainability ecolabel depends not only on the origin from a sustainable source, but also on the supply chain committing to pay for use of the label so as to guarantee full product traceability to the final buyer (i.e., avoid mislabelling and fraud). Thus, fishers that want the benefits of the MSC ecolabel on consumer-facing products might change what markets they sell to in order to benefit from a fully certified supply chain. In some cases fishers may sell only part of their catch with an ecolabel, for instance the part of their catch that has a higher end product line. Some may not sell with the ecolabel at all if the demand for sustainable product is at the supply chain business-to-business level, not the consumer end [49].

Recently, there has also been increasing evidence that drivers for fisheries sector to become certified can also be of a social nature [12], and often the ability to remain certified can depend on cooperation from management institutions willing to facilitate necessary research or policy improvements [12]. Thus it seems that a more complex combination of social, economic and political drivers [27, 50] may influence the decision to become or remain certified [12].

In this study, we use semi-structured interviews with the aim of better understanding the fisheries sector participation in certification schemes (the supply side as well as first buyers in the supply chain, where a certified supply chain is present) and the benefits and costs of MSC certification. The perceptions of benefits and costs are likely to influence stakeholder participation and the supply of certified fish. We focus mainly on the economic, social and institutional impacts (but also cover some of the environmental impacts) from the perspective of key informants for different stakeholder groups in seven different MSC certified fisheries that operate in (or from) West Australia (WA).

## 2 Methods

### 2.1 Case study description

In 2012, the WA government provided the State's commercial fisheries with AU$14.56m to support MSC certification costs [51] and to help fisheries with the scientific support to make improvements needed to meet certification requirements. $6.56m of the total covered the pre-assessment and the initial full assessment process for participating fisheries and the initial surveillance audits after achieving full assessment. This seed funding provided by the State government was driven by a range of expected benefits across both government and industry. In the project scoping phase that took place prior to the commitment of state funds, a broad set of expected outcomes were identified. One of the expected outcomes of this jurisdictional commitment to third party certification included credible and defendable sustainability claims with regard to industry practices and government stewardship. Other expected outcomes were, securing and maintaining access to new and established markets, security of access to fishing grounds, and encouragement for investment in regional fisheries. In addition, it was also hoped that by making the financial commitment to MSC, it would make sustainability data for fisheries publicly available to different organisations that make frequent requests for this data (i.e. retail, non-government organisations, and the Australian government) through the MSC certification reports. This would thus reduce the WA Department of Fisheries’ costs associated with providing this data which would now be provided in a standardised and globally recognised format.

The State government’s third-party certification process was carried out in partnership with the WA Fishing Industry Council (WAFIC) and Recfishwest, which are the representatives for bodies representing the commercial and recreational fishing sectors, respectively, in WA. Both representative bodies are formally recognised by the Government of WA as the main sources of industry advice for the recreational and commercial sector respectively. Therefore, these bodies have direct input into WA fisheries’ management process, compliance issues, research and the MSC certification process among other priorities. The government’s decision to provide funding for MSC certification meant that the (financial) barrier to certification was lowered by reducing the upfront certification cost, as well as the cost of seeking the technical and institutional support for any needed improvements. Participation by WA commercial fisheries in the full MSC assessment process remains a voluntary step, recognising that some fisheries may not choose to proceed to full assessment.

**Fig 1:**
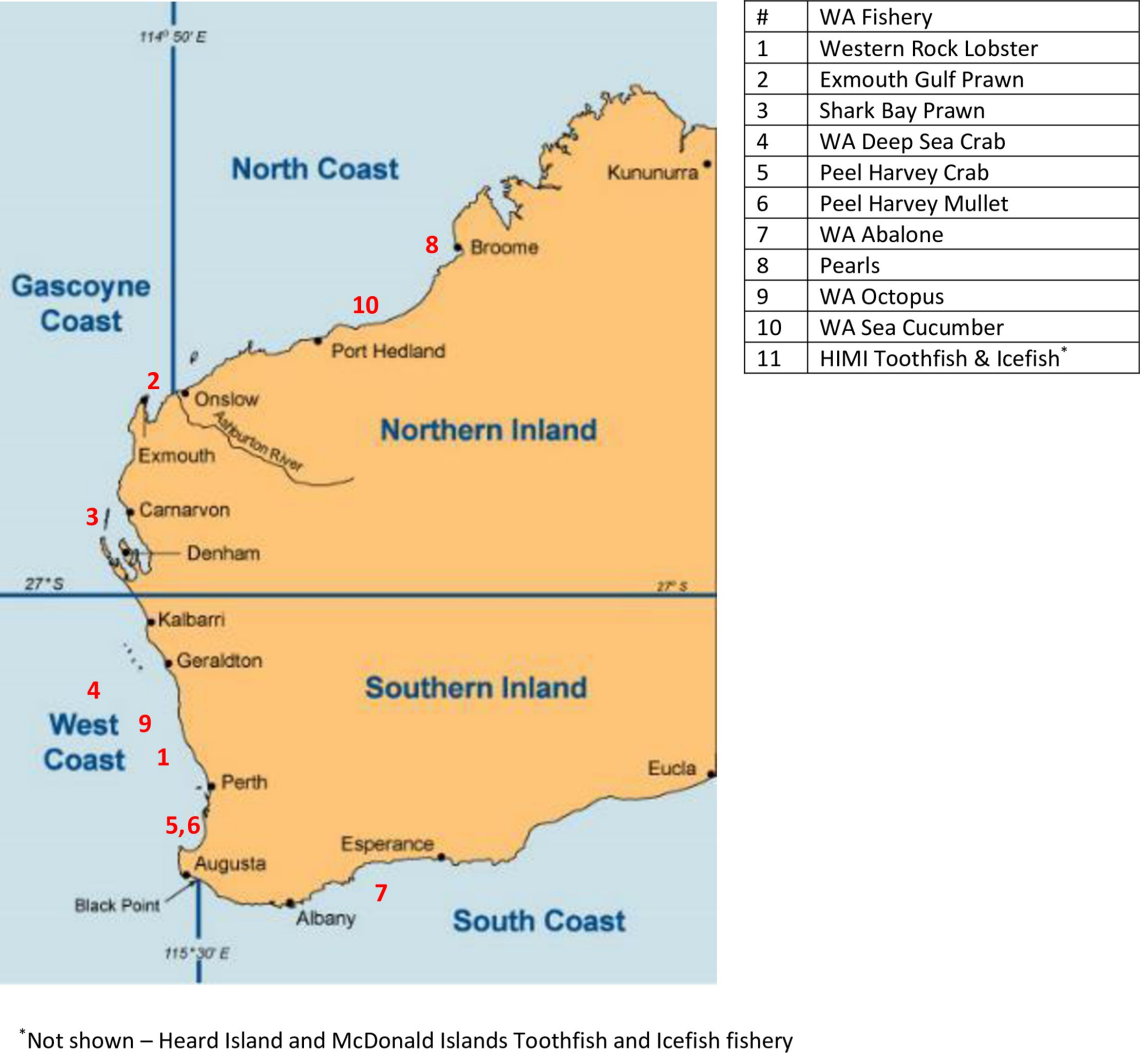
The four Western Australian marine bioregions and the approximate fishing location of the MSC certified fisheries in Western Australia.

The western rock lobster fishery did not financially benefit from the government’s third-party certification program as it was already MSC certified. In 2000, the western rock lobster fishery was the first in the world to attain MSC certification. The western rock lobster fishery was re-certified for the fourth time in 2017. The Exmouth Gulf and Shark Bay prawn fisheries were the first to be MSC-certified under the government’s funding program. Currently certified fisheries include: the Peel-Harvey Estuary blue swimmer crab fishery (the first fishery to be certified with a recreational and commercial component); the Peel-Harvey Estuary sea mullet; the West coast deep sea crab fishery; pearl oyster (the first 'gem fishery' to be MSC certified); and West and South coast abalone (Roe’s, greenlip and brownlip abalone). These fisheries are the subject of this paper (Fig 1). Since the qualitative stage of this research was completed, two further WA fisheries have achieved MSC certification: the Western Australian octopus and Western Australian sea cucumber, but these are not included in the present research. We include one MSC certified Commonwealth fishery: The Heard Island and McDonald Islands Patagonian toothfish and mackerel icefish fishery. This fishery was MSC certified in 2004 and because it is not a WA state fishery, it was not eligible for government funding.

At the time of this current study, the State government funding scheme had concluded with all 50 of the States fisheries now being MSC pre-assessed. 10 of those fisheries have gone on to achieve and currently maintain MSC certification. The portfolio of Western Australian MSC certified fisheries represent 90% by value of the state’s wild catch fishery landings. For those WA fisheries yet to decide on the voluntary transition through to MSC full assessment, State government funding remains available to support the cost of the initial fishery improvements and the initial MSC audit costs in order for interested fisheries to participate in the MSC program. Unless the government makes additional funding available, those fisheries that have benefitted from the initial funding support will now incur ongoing certification costs for annual surveillance audits and the five-yearly reassessment process.

### 2.2 Management of Western Australian fisheries

WA fisheries are managed through a series of legislated plans, regulations, orders and licence conditions, using a combination of input and output controls (i.e., total allowable catch (TAC), seasonal closures, and size limits). The plans are developed in conjunction with industry, Industry representative bodies, industry associations, and community groups [52].

A total of seven fisheries are the subject of this research (Fig 1), six of which are managed by the WA State Government (Table 1).

**Table 1:** Details of Western Australian MSC certified fisheries.

Western Australian Fishery	Target Species	Beach Value (2017)	Primary Management System	Number of vessels in MSC certificate	Catch (2017)	MSC Certification Date
**Western Rock Lobster**	Western Rock Lobster (*Panulirus cygnus*)	$386m	TACC	234	6,400t	2000
**Exmouth Gulf Prawn**	1. Western King Prawn (*Penaeus latisulcatus*)	$10m	Input controls	6	713t	2015
	2. Brown Tiger Prawn (*Penaeus esculentus*)					
	3. Endeavour Prawn (*Metapenaeus endeavouri*)					
**Shark Bay Prawn**	1. Western King Prawn (*Penaeus latisulcatus*)	$26.4m	Input controls	18	1,608t	2015
	2. Brown Tiger Prawn (*Penaeus esculentus*)					
**Deep Sea Crab**	Crystal (Snow) Crab (*Chaceon albus*)	$6.3m	TACC	3	164t	2016
**Peel Harvey Crab**	Blue Swimmer Crab (*Portunus armatus*)	>$1m	Input controls	11 (commercial)	75.2t^1^42.9t^2^	2016
**Peel Harvey Mullet**	Sea Mullet (*Mugil cephalus*)	>$1m	Input controls	11	127.1t	2016
**Abalone**	1. Roe’s Abalone (*Haliotis roei*)	$5.89m	TACC	30	147t	2017
	2. Greenlip Abalone (*H*. *laevigata*)					
	3. Brownlip Abalone (*H*. *conicopora*)					
**Pearl Oyster**	Silver Lipped Pearl Oyster (*Pinctada maxima*)	$53m	TACC	6–10	468,573 (shell count)	2017
Octopus^*^	*Octopus (Octopus aff*. *tetricus)*	*$2*.*5m*	*Input controls*	*23*	*257t*	*2019*
Sea Cucumber^*^	*1*. *Sandfish (Holothuria scabra)*	*>$1m*	*Input controls*	*6*	*135t*	*2019*
	*2*. *Redfish (Actinopyga echinites)*					
**Heard Island & McDonald Islands Toothfish & Icefish^#^**	1. Patagonia Toothfish (*Dissostichus eleginoides*)	*Confidential*	TACC	5	3525t	2006 (icefish) 2012 (toothfish)
	2. Mackerel Icefish (*Champsocephalus gunnari*)					

* MSC certified in 2019 but not included in the study.

^#^ Heard Island and McDonald Islands Toothfish and Icefish is an Australian Commonwealth fishery.

^1^ Commercial catch.

^2^ Estimated recreational catch (by boat).

The largest is the western rock lobster fishery that is also the largest WA fishery in volume and value, and one of the most valuable single-species fisheries in Australia. Eight species of rock lobster are found off the coast of WA. However, virtually all lobsters caught are the Western rock lobster, found up to 60 km off the coast between Augusta and Shark Bay (see Fig 1) [52].

WA’s second most valuable fishery, WA’s pearling, is a quota-based dive fishery, operating in shallow and sheltered coastal waters along the North-West Shelf [52]. More than half of Australia’s blue swimmer crab fishery are caught commercially in WA, and it is a popular recreational fishery. Recreational fishing in Western Australia is a popular activity with an estimated 700,000 of the States 2.6 million people participating. The exact number of recreational fishers that target blue swimmer crab is unknown, but recreational boat-based catches were estimated to be over 50 tonnes in 2017–2018 [53]. In 2014, the Cockburn Sound crab fishery was closed indefinitely [52], due to indices of slow recovery and seasonal closures apply to various locations in WA [54]. Since 1999, the main target species for the Deep-Sea Crab trap fishery has been the crystal crab [52]. Operating in waters deeper than 500m off the west coast of WA traps are operated in long-lines. The Exmouth Prawn fishery, located in Exmouth Gulf in WA, targets different short- lived, fast-growing prawn species that have variable environmentally driven recruitment. The fishery commenced in 1963 and currently has 15 managed fishery licences, all of which are held by a single licensee [52]. The Shark Bay prawn fishery operates within the Gascoyne Coast Bioregion of WA. Shark Bay is a World Heritage Area for its ‘natural heritage values’ [55]. The prawn fishery in Shark Bay works under a limited entry system and is currently the highest producing prawn fishery in WA [56]. Patagonian toothfish and mackerel icefish are targeted in areas of the Australian fishing zone adjacent to Heard Island and McDonald Islands. The fishery is managed by the Australian Fisheries Management Authority (AFMA). They are predominantly caught by demersal longline; however, trawl gear is also used to harvest mackerel icefish [52].

### 2.3 Survey design

The survey applied to key informants for MSC certified fisheries in WA was based on a pre-existing survey template that was implemented in the U.S. West Coast albacore tuna fishery, the South Brittany sardine fishery, and the Portuguese sardine fishery in 2017. The initial piloting of the survey in these fisheries, focussed on mainly on the processing industry, and is the topic of another paper. The study conducted in WA was intended to further assess if the survey could be implemented more broadly to MSC fisheries, and increase the number of different fisheries being compared, as part of a long-term plan to gradually build up a large-scale dataset of sites across the world.

The original survey was co-developed through a series of workshops and consultations with MSC staff and external academics [57]. The aim of the overall project was to develop a survey that, through a ‘rapid assessment’ approach that is systematically reproducible and standardized across different fisheries in different parts of the world, could identify differences and commonalities in observed socio-economic effects of MSC certification on benefits and costs for fisheries and supply chain (at least first buyers), changes in employment and supply chain structure and stakeholder relationships. The purpose was for both MSC impact evaluation and internal learning about the effective pathways within the MSC Theory of Change [57]. As a member of ISEAL Alliance, the MSC is required to implement best practices for Monitoring and Evaluation, including assessment of the indirect or unintended social effects of their environmental certification [58].

To gain a deeper understanding of how desired behavioural change towards sustainability is achieved, the MSC’s declared Theory of Change [21] was applied to guide the design of the survey. The Theory of Change [59, 60] helps understand and describe how and why behaviour changes may arise. MSC uses its Theory of Change as a basis for the design of the program, as it maps out how MSC activities and interventions lead to achieving their sustainability goals. More specifically, the Theory of Change posits that market demand for seafood sustainability provides added benefits for those products that can demonstrate, through the MSC ecolabel, that they meet environmental sustainability best practice. As more ecolabeled products appear on the market, this increases public awareness and recognition, in turn driving demand. Competition with ecolabeled products and the desire for other benefits of the certification eventually incentivise fisheries that are less sustainable to invest time and resources in the transformative change that is required to drive improvements, including partnerships and interactions with other players (e.g. supply chain actors, managers, environmental NGOs) [61]. Even though the pathways to change were mapped in an ‘outcomes framework,’ a survey to test of whether the assumed pathways and result chains actually occur as posited by the MSC had not been previously developed. In addition to testing the expectations based on the Theory of Change, this monitoring approach also checks for unexpected (positive or negative) consequences.

Three key themes are included in the survey: i) economic costs and benefits of certification (e.g., price premiums, new or retained market share); ii) changes in the supply chain driven by producers selling to a certified supply chain in order to use the ecolabel (e.g., narrowing of the supply chain) and/or reach new markets, and potential consequences to distribution of revenue across the supply chain (e.g., change in price bargaining power of suppliers or buyers), and/or changing product form, with consequences on employment structure or redistribution of benefits across actors in the supply chain (especially first buyers); and iii) interactions and conflict between groups of certified and uncertified harvesters and partnerships among industry groups, managers and NGOs that were developed through the certification process [57].

To accommodate context specific issues, minor changes were made to the original survey instrument before it was applied in WA. Ethics approval was obtained through CSIRO (093/19) and consent forms were signed by all participants.

### 2.4 Survey implementation

A total of thirty-three key informant interviews were implemented in WA in early 2019. Thirty interviews were carried out face-to-face and three interviews were by phone. Key informants were selected due to their professional engagement with MSC fisheries in WA, and therefore their ability to provide rich data on MSC and a particular fishery, not to obtain a representative sample of the State’s fisheries. A snowball sampling strategy was then employed where the initially selected key informants were asked (or they volunteered) the names of other persons to interview. Attempts to avoid sampling bias were also addressed by checking the appropriateness and comprehensiveness of selected key informants with individuals who had domain knowledge but were no longer involved with WA fisheries. Therefore, ensuring confidentiality at the beginning of each interview was key for exploring negative perceptions on MSC certification and its outputs.

Participants were initially contacted by email, and appointments were made for a time and location of their choice. The survey duration was between 30 minutes and 1 hour (with 2 survey taking more than one hour). The interviews were taped with the approval of the participants and in accordance with Ethics approval guidelines. The surveys were implemented by the first author together with one or more of the co-authors. Where none of the co-authors were available the interviews were carried out by the first author alone (i.e. the phone interviews and 3 face-to-face interviews).

Even though the aim was to engage stakeholders from different groups and across the different MSC certified fisheries, it proved difficult to gain participation from some groups (i.e. two smallest groups in Table 2) and the number of informants was also limited by the small number of individuals that were sufficiently knowledgeable of the processes we wanted to investigate. The implications of low participant numbers for some stakeholder groups are acknowledged and accounted for in the data analysis and presentation of the results. The principle of saturation [62] was used to provide an assessment of the adequacy of the number of key informants interviewed.

**Table 2:** Survey respondent numbers by stakeholder group and MSC certified fishery.

Row Labels	All west Australian MSC fisheries*	Abalone	HIMI toothfish and icefish	Pearl oyster (wild collection)	Peel-Harvey Estuary blue swimmer crab& Sea mullet	Shark Bay & Exmouth Prawn fishery	West coast deep sea crab	Western Rock Lobster	Total Resp
Government scientist	1	1		1	2	1	1	2	9
Government manager	3			1		2			6
Fisher		1			2			1	4
Fishing company			3			1			4
Fishing association**					1	1		1	3
Processor						1	1	1	3
Industry association (WAFIC)	2								2
Non-Government Organisation					1				1
Academic scientist								1	1
**Grand Total**	**6**	**2**	**3**	**2**	**6**^**#**^	**6**	**2**	**6**	33

* Respondents in this group indicated they worked across all the MSC certified fisheries in WA.

** A respondent belonging to a Fishing association was a key informant only focusing on one fishery whereas the industry association informants were discussing all fisheries in Western Australia.

^**#**^ Two respondents represented the recreational fishing sector.

A total of 33 key informants (further referred to as respondents) were interviewed. 34% of the respondents [10] were female, with most female respondents (8 out of the 10) in management and science. The remainder were male. The largest group were government related professionals [15] including scientists [9] and government managers [6]. Industry respondents were the next largest group represented by 14 respondents: fishers [4], fishing company representatives [4], processors [3], and fishing association representatives [3] (Table 2). The remainder of the respondents represented industry associations [2], academic scientists [1], and NGOs [1]. Results for the latter three categories cannot be revealed in detail due to low numbers and confidentiality considerations. Responses for industry associations, academic scientists, and NGOs are grouped into an ‘other’ category from here onwards.

### 2.5 Data analysis

The quantitative survey questions were entered in a CSV file and analysed in R [63]. The rich narrative around open questions (the qualitative data) was coded and analysed thematically. The qualitative interview data were derived from question about the drivers for MSC certification (see question 10 in S1 File) and regarding the impacts of MSC certification (questions 30 to 34 in S1 File).

For the thematic analysis, the original recordings were coded and analysed; however, the recordings were not transcribed. Recordings were analysed using ‘bottom up’ (inductive) and iterative coding followed by thematic analysis, where the recordings were coded according to the ideas and meanings that were present in the respondent answers to the questions. The first author was responsible for the coding but the themes and results were shared and discussed by some of the co-authors who assisted with the interviews to ensure consistency and accuracy. The result was a hierarchical structure of themes and sub-themes through multiple rounds of listening to the interview recordings.

Key themes on the impacts of certification in different fisheries were further explored. The themes were typified according to whether the MSC certification impact was perceived to be negative or positive. However, the interview style may have encouraged more positive than negative impacts to be volunteered which is acknowledged in the data interpretation presented in the next section.

## 3 Results

On average, the 33 survey respondents had 14.5 years’ experience in fisheries. In particular, the individuals directly involved in the sector (i.e., fishers and fishing company representatives) had a long history of experience, and presumably a considerable amount of domain knowledge, which was captured in the survey. Fishing company representatives had been in their profession the longest (on average 22 years) followed by fishers who had been involved for around 19 years (on average). Processors had the shortest period of experience (8 years average). The other stakeholder groups had between 11.5 and 15.5 years of experience.

The catch sector in WA does encounter a lot of multi-generational engagement with many fishers undertaking a career in fishing through family ties to the industry. This tends to lead to high levels of experience (especially with those representing industry interests at the executive level) even with younger operators.

Due to the capacity limitation in not being to interview each active participant in a fishery, the research team tried to capture stakeholder views by engaging those who often represented industry interests at a fishery or sectoral level. These stakeholders are frequently in these roles due to their experience in working on fishery-specific issues which is why the catch sector respondents may have a higher than expected level of industry experience.

### 3.1 Drivers for obtaining MSC certification

**Fig 2:**
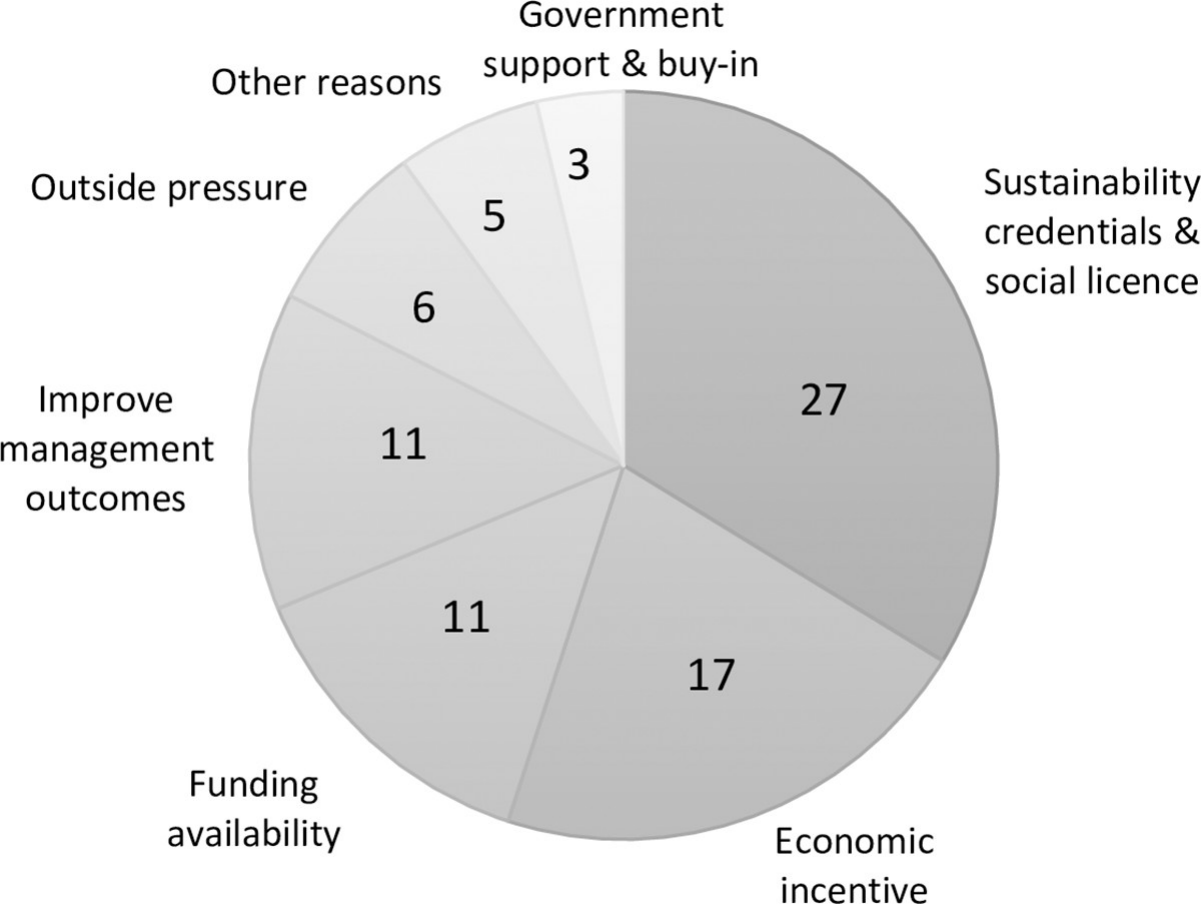
The number of mentions of the drivers (in the top three) leading to fishery MSC certification.

The reasons for gaining certification (the drivers) can be illustrative of people’s expectations of the impact (benefits or lack thereof) of certification. We asked respondents for the top three reasons they believed their fishery sought MSC certification (Fig 2). Some respondents chose to list only one or two drivers.

Respondents mentioned 80 different drivers. The most frequently mentioned reason for getting MSC certification was to improve sustainability credentials and to gain or maintain social licence (including being seen to be a world leader) (see S1 Table for more detailed descriptions of the labels in Fig 2). Economic incentives were the second most commonly mentioned reason for becoming certified, which included market access, marketing advantages more generally, and product differentiation. Perhaps surprisingly, obtaining a price premium was only mentioned once among the top three reasons for certification. Fisheries that did not receive funding to become certified compared to those who did found sustainability credentials and social licence (43% versus 27% respectively), and economic incentives to be more important (43% versus 16% respectively).

The availability of government funding to become certified (previously discussed in section 2.1) and to improve management outcomes (mainly environmental outcomes) were the next most frequently mentioned (11 times each). A reduction or lowering of the financial barrier to become certified through government funding was thus important for eligible fisheries. The western rock lobster fishery and the toothfish and icefish fisheries were both already certified prior to 2012 when the funding program was initiated. Moreover, the latter is a Commonwealth managed fishery and was thus not eligible in the first place.

### 3.2 Benefit versus costs

Respondents were asked to weigh up the benefits and costs of MSC certification, including non-monetary benefits and costs. The costs include those that are incurred prior to certification (called transition costs), like making changes to meet the MSC standard (e.g., catch methods, installing bycatch exclusion devices, or developing data streams). The costs also go beyond the direct costs associated with certification (i.e., audits) and include indirect costs (i.e., monitoring data and extra time required for reporting). Monetary benefits are generally thought of as economic benefits such as increased revenues from price premiums. However, some of these indirect costs can be non-monetary. Non-monetary benefits are often associated with social, institutional, or environmental aspects. Social benefits include, for example, improvements in social acceptability of the fishery, better relationships, and/or improved communication between the fishing sector and the managers of the fishery. Institutional benefits include, for instance, improved stakeholder consultation processes. Environmental benefits may include reduced bycatch, or improved outcomes for endangered species.

**Fig 3:**
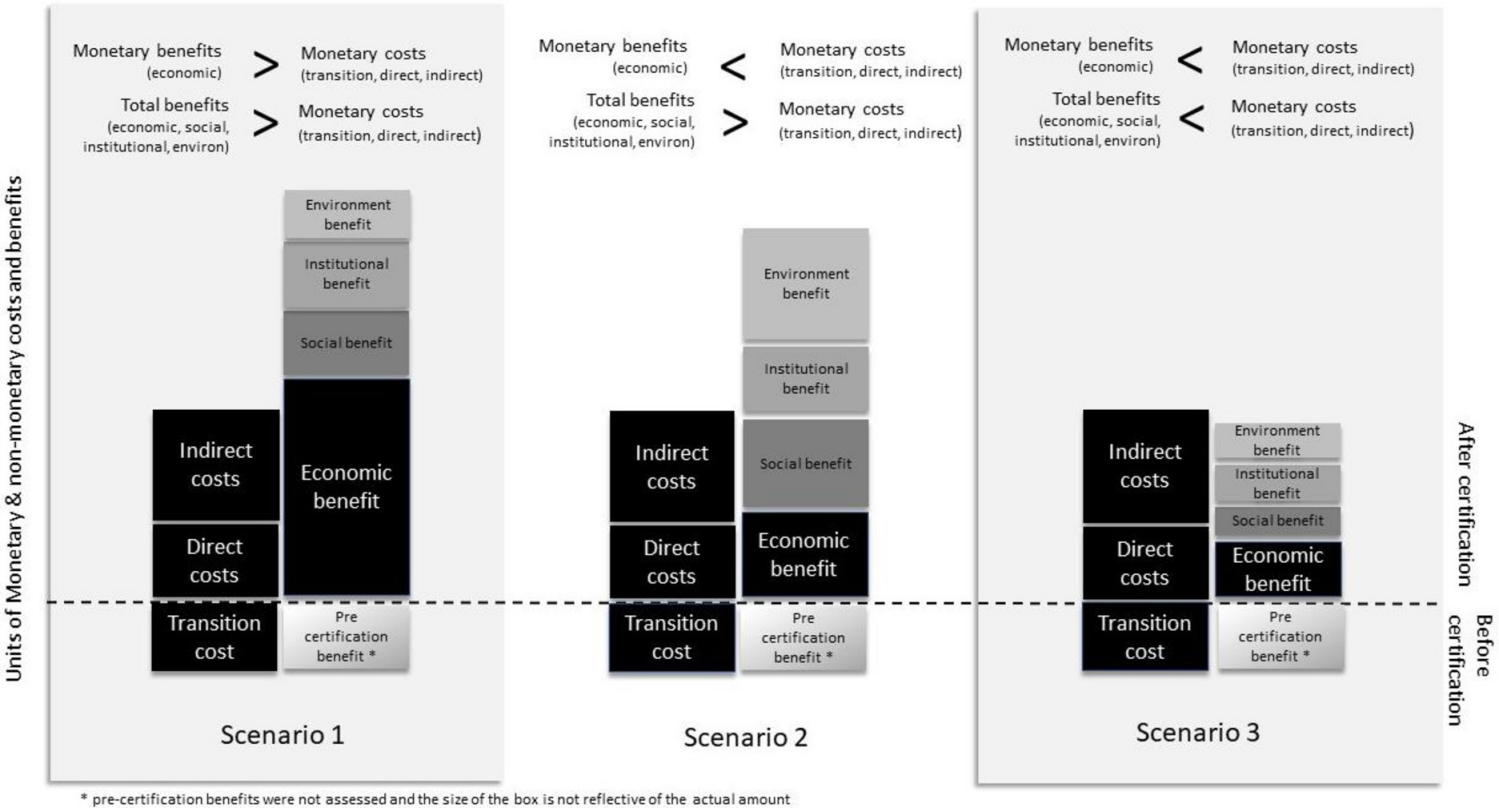
Three scenarios showing the hypothetical relationship between the benefits and costs of MSC certification to a certificate holder/group. The economic benefits (monetary benefits) are shown in black and the monetary costs (which includes transition, direct, and indirect costs) also shown in black. The non-monetary benefits are shown in different shades of grey and include social, institutional and environmental benefits. The transition costs are incurred before certification, shown below the dashed line. Presumably some benefits may be incurred before certification (shown in the grey dashed box below the dashed line), but this was not assessed in this research. In the first scenario the monetary (economic) benefits are greater than the monetary costs. In the second scenario the economic (monetary) benefits do not outweigh the monetary costs, but the total benefits outweigh the monetary costs. In the third scenario the hypothetical total benefits do not outweigh the monetary costs of certification.

Just over half of respondents indicated the benefits of MSC certification outweighed the costs (19 out of 33). Of these 19 respondents 10 were from the fishing sector and 9 from management and science. However, 10 of the respondents (6 respondents from the fishing sector and 4 from management and science) who indicated that the benefits were greater than the costs stipulated that this was not the case if only monetary benefits (economic benefits) were considered. In the case where only economic benefits were considered, most of these respondents suggested that the costs would outweigh benefits instead. In other words, the benefits were only greater than the costs when the non-monetary benefits of certification were included (Scenario 2 in Fig 3). The benefits of MSC certification of Western Australian fisheries do not currently result in direct ‘money in the pocket’ of the fisher or actors in the supply chain. The benefits are of a more non-monetary nature.

Five respondents (2 respondents from the fishing sector and 3 from management and science) explicitly indicated that the benefits only outweighed the costs because the government had co-invested in getting the industry certified. As previously indicated, the third most mentioned driver for certification in Western Australian fisheries was the fact that the government paid for the pre-assessment, the full assessment, and the first annual audit. Therefore, respondents may have only included the indirect costs of certification in their benefit:cost assessment because the direct and transition costs were not incurred by their fishery.

Nine respondents indicated that the economic benefits clearly outweighed the total costs (scenario 1 in Fig 3). The economic benefits for the fisheries represented by these respondents were largely attributed to market access and price premiums. Only two fisheries that were the subject of this study were selling (some or all of) their product with the MSC label and were able to potentially capture a price premium in the market. The supply chains of these fisheries were more vertically integrated than other fisheries. Given this, integrated supply chains appear to be an enabling factor for using the label because it is easier to have a fully certified supply chain that is thus able to use the ecolabel on consumer-facing products.

Nine respondents (3 respondents from the fishing sector–but all 3 representing different fisheries—and 6 from management and science) indicated that they were unsure about the benefit:cost ratio. Some indicated that they were unsure exactly how much value (benefit) they had received or that it was not clear yet if the benefits outweighed the costs. Respondents representing fisheries where certification was funded by the government had thus far not incurred any costs nor seen any benefits. Their benefit:cost ratio will only become truly apparent when they must pay for their first audits and re-certification.

Five respondents (3 respondents from the fishing sector and 2 from management and science–all five representing different fisheries) felt that the costs were greater than the benefits and that this was particularly the case because there was still no evidence of a thriving marine environment (i.e., there was a perceived absence of environmental benefits).

There was no information collected in the survey to directly assess if respondents indeed consider the non-monetary side of the ledger in their assessment of the benefit:cost ratio (it is only known for those who volunteered this information). To aid in that understanding key informants identified the types of impacts that MSC certification had on the different fisheries.

### 3.3 MSC impacts

Respondent perceptions of the economic, social, environmental and institutional impact of MSC certification were either positive, negative or undefined where there had been no impact. On average each survey respondent indicated 12 different impacts (n = 380). The qualitative responses regarding impacts were coded with a high-level impacts theme that separated the impacts into social (133, 59 plus 74 in Table 3), economic (112), institutional (91), or environmental (44) (a more detailed breakdown is shown in S2 Table).

**Table 3:** MSC impact domain (economic, social, environmental and institutional) and the direction of the impact (positive, negative or otherwise) for the fishing sector (processors, fishing companies, and fishers*) and management & science (government managers and scientists and academics*).

Impact domain of MSC certification	positive impact	negative impact	no impact	Grand Total**
Economic	22	6	36	64
Social	43		12	55
Environment	22	1	4	27
Institutional	34	1	5	40
**Fishing sector (total)**	**121**	**8**	**57**	**186**
Economic	13		29	42
Social	53	4	13	70
Environment	13	2	2	17
Institutional	32	7	6	45
**Management & science (total)**	**111**	**13**	**50**	**174**

* individual categories cannot be revealed for confidentiality reasons.

** there were 5 mentions of limited impacts with no mention of direction, or where it was unclear what the direction of the impact was (15 mentions). These have been left out of the table.

The respondents from the fishing sector (processors, fishing companies, and fishers) mainly focussed on the economic impacts of MSC certification (68%, 38%, and 36% of these group’s impact comments respectively). Government scientists largely focused on the social impacts of MSC certification (43% of this group’s impact comments). Government managers mainly identified institutional (39% of impacts comments) and social impacts (35% of impact comments). The impact comments of the fishing associations were mainly institutional (38%) and social (44%). There were no significant differences in the answers between the respondents from fisheries that received funding and those that did not.

#### 3.3.1 Direction of MSC impact

**Fig 4:**
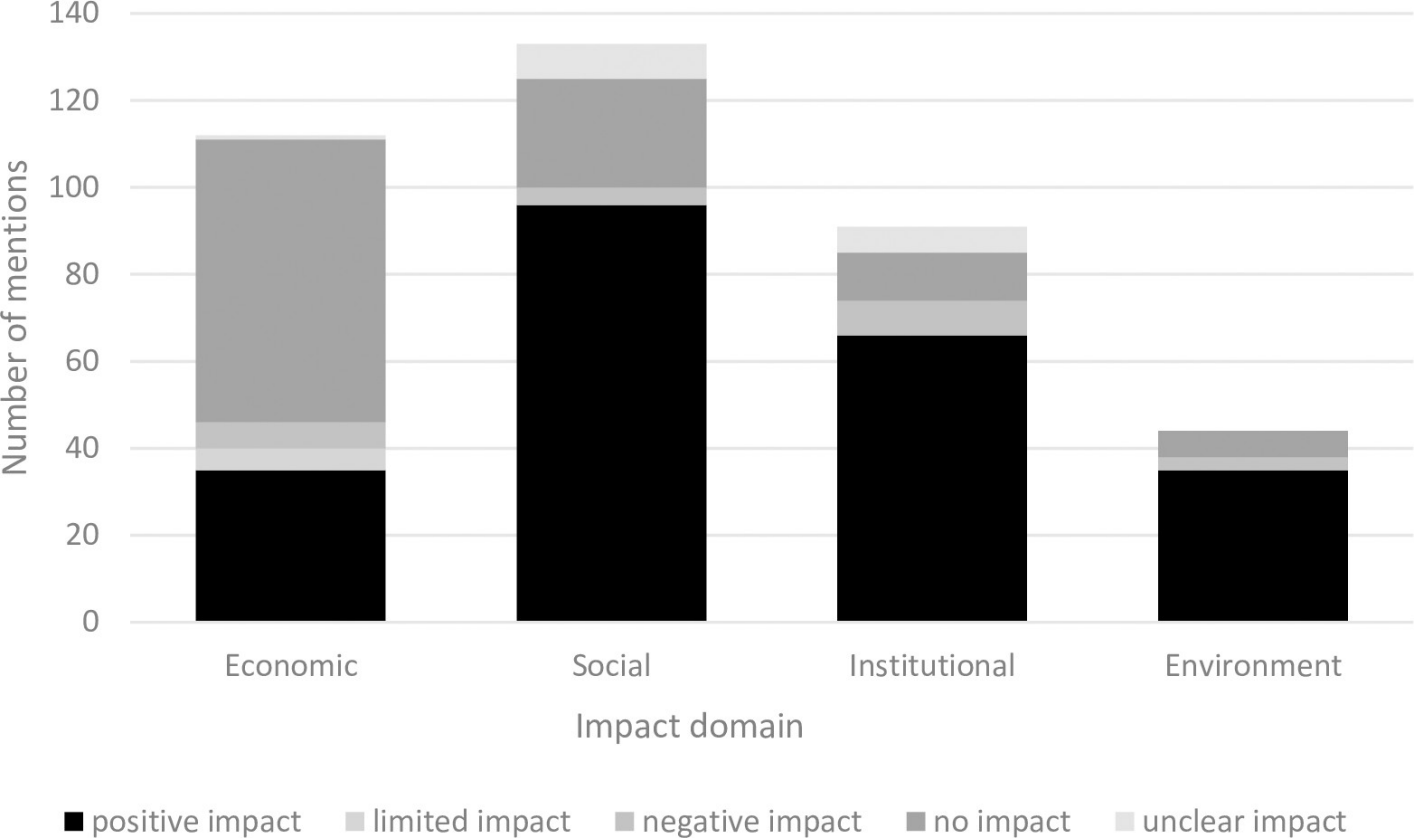
Economic, social, institutional, and environmental impact of MSC certification and the types of impact effect.

Respondents tended to focus on the positive impacts of certification. Perhaps, the focus on the positive side of the impacts may be an artefact of responder expectations because the research was funded by the MSC although implemented by an independent researcher. Every attempt was made to ensure the respondents were aware that the interviewer sought to find out about both negative and positive impacts. A total of 232 comments were indicative of a positive impact of MSC certification (61% of the total number of comments), 28% of comments indicated the MSC certification had no impact (107), and 6% of comments (21) indicated a negative impact from MSC certification (Table 3 and Fig 4). Just over 5% of comments (20) indicated that the positive or negative impact had either been limited, or the impact was not clear.

The economic impacts were largely perceived not to have eventuated (no impact). The social, institutional, and environmental impacts were largely perceived to have been positive.

#### 3.3.2 Economic impact

Economic aspects were the second most important driver for pursuing certification (Section 3.2). In evaluating the impacts (as opposed to the drivers), it became evident that many comments indicated that the expected economic impacts had in fact not materialized (i.e., 58% of comments indicated there was no perceived economic benefit of MSC certification). In other words, there was a clear economic driver for initially pursuing certification, but the (expected) economic impacts are currently not apparent in many of the certified WA fisheries. The lack of economic benefits was predominantly attributed to a lack of brand recognition (related to a lack of market demand) in both domestic and export markets (20 comments, 31%), the absence of price premiums for certified product (10 comments, 9%), and the absence of the need for certification to access current (mainly Asian) sale markets (9 comments, 8%).

QUOTE: “I was quite surprised that the fishers wanted to go through MSC given they mainly sell to China … definitely no financial benefit.” (Respondent #6, government scientist)

QUOTE: “Pricing has gone up quite materially but not as a consequence of MSC.” (Respondent #2, processor)

The perceived negative economic impacts of MSC certification were related to increased costs. These increased costs included expenses related to chain of custody certification [12] and higher business costs more generally. As illustrated in the comment below, the additional paperwork and (re)certification requirements are increasing the complexity of the traditional business model where the sale of product was more straightforward and required less record keeping.

QUOTE: “It has become harder and more expensive [for fisheries] to do business [*because of MSC requirements*]” (Respondent #24, processor)

Even though a large proportion of comments were centred around the lack of, or negative economic, impact of MSC certification, 35 comments (31%) indicated there had been some positive economic impact (22 of these positive comments were made by the fishing sector). Ironically, the positive economic impacts that were mentioned were the direct opposite of the negative impacts. For instance, respondents indicated there had been a positive effect in terms of market access and price premiums, as well as marketing advantages. These positive impact on market access and price impacts were particular to certain fisheries (i.e., HIMI toothfish and icefish and Australian prawn fishery’s).

QUOTE: “Branding—ecolabel helps, looks good, and gives access to different markets.” (Respondent #27, fishing company)

Only those few fisheries in WA that pay logo-licencing have been able to capture a price premium from the MSC label. The relationship between selling product with the MSC label, vertically integrated production systems, and control over the supply chain, may explain these positive economic impacts (price premiums and market access) for these few fisheries.

#### 3.3.3 Social impact

The social impacts of MSC certification were mainly positive (Table 3). Around a quarter of the positive social impacts were related to obtaining or retaining social licence, followed by increasing knowledge levels (predominantly about the environmental impact of the fishery) (17%) and improvements in communication (16%) between stakeholders generally, and between the government and fishery participants more specifically. Stakeholder collaboration on solving issues arising in their fishery (13%) had also improved.

QUOTE: “Things have recovered and MSC has been used as social licence tool.” (Respondent #33, fisher)

However, on the issue of social licence and collaboration, some strong alternative or opposite views were expressed by both the fishing sector and managers and scientists. The opposite view was particularly strong in one fishery, but due to the small number of observations we cannot reveal the name of the fishery in question for reasons of confidentiality. Regarding social licence there was a perception that there was little evidence (or it was unclear or not yet determined) that social licence was either obtained or retained.

QUOTE: “Small fishery along a long piece of coast—fishing in small towns—but there was not a lot to gain in these small towns where social licence is concerned.” (Respondent #7, government scientist)

QUOTE: “Still working on the social license, but people will always think that the commercial fishers do not belong there.” (Respondent #29, fisher)

In relation to stakeholder collaboration, respondents in one particular fishery indicated that it had in fact deteriorated because of MSC certification rather than improved. These respondents indicated that the opportunities for collaboration in research projects had worsened.

QUOTE: “[*certification*] has created anxiety—exacerbated difference between academic and gov/department scientists” (Respondent #32, other)

Only 19% (Table 3) of comments indicated that there had been no social impact.

#### 3.3.4 Environment

The environmental impact of MSC certification were predominantly perceived to have been positive (80% of environmental comments). The positive impact was mainly around the overall fishery impact on the environment, as well as environmental management by these fisheries.

QUOTE: “Habitat work done in Shark Bay (this would not have happened without the MSC).” (Respondent #15, other)

QUOTE: “industry has implemented sea snake bycatch and handling.” (Respondent #21, government manager)

Some of the negative comments questioned the MSC environmental rules, which overall led to worsened environmental outcomes in their opinion.

QUOTE: “Expected more scrutiny from the MSC. The biomass is clearly under threat and the MSC is too conservative.” (Respondent #24, processor)

Many mentions were made of environmental improvement that were due to MSC requirements including benthic mapping, snake handling knowledge, and selectivity through the use of bycatch exclusion devices. Snake handling was categorised as environmental because it concerns protected species, but this also has a health and safety aspect, and could also be categorised as a social impact.

#### 3.3.5 Institutional

The institutional impacts of MSC certification were mainly perceived as positive (73% of the comments—Table 2). There were positive impacts on transparency (of management and fisheries process) [17], gains in political influence [13], and increased funding (resource) availability [11,12, 64].

QUOTE: “Information was there if the fishers wanted it—and they could have contributed if they wanted it (they are satisfied).” (Respondent #1, fisher)

QUOTE: “They could get greater services from the department—during the pre-assessment phase they became aware of that.” (Respondent #6, government scientist)

12% of comments indicated no institutional impact and 9% indicated a negative impact (Table 2). Again, some respondents perceived negative impacts on the same issues that others had perceived there to be a positive impact. For instance, they perceived there to be a negative influence on transparency and on the management process more generally. In particular, that the communication and transparency of fishery information from the government department (currently called Department of Primary Industries and Regional Development, or DPIRD) that is submitted for MSC purposes should be more readily available to the industry.

QUOTE: “Risk when industry takes over the assessment audit–they have to communicate better.” (Respondent #12, government scientist)

QUOTE: “[*following the rules*] caused us a lot of work—didn't have the resources for it sometimes.” (Respondent #20, government manager)

QUOTE: “Can’t kick the sustainability issue back to government because they should already be doing it. So the data needs to be accessible and transparent -at the moment it is not.” (Respondent #32, other)

### 3.4 Sources of conflict and confusion

The MSC pre-assessment and certification follows several stages and processes. Stakeholders involved in the certification process may be familiar with at least some, if not all, of the requirements that are part of the process. In the beginning respondents did, however, find some parts of the process confusing, mainly around some specific definitions such as 'units of certification’. For example, each ‘unit of certification’ in a MSC certificate is awarded for a very specific group of vessels operating with a particular gear on a specified species, but the effects of other vessels fishing on the same species also need to be considered, even if they aren’t involved in the certification, as part of the same ‘unit of assessment’ so as to ensure a full evaluation of the exploitation pressures that the certified fishery is subject to. This leads to confusion about what needs to be assessed, but also provides an incentive for all operators fishing in the same area to partner up and share the costs of the certificate. Even though most confusion was resolved without further consequences, confusion that arose later in the process around, for instance, changes in bait requirements due to a new version of the Standard led to some tensions between MSC, the Department of Fisheries (DPIRD), and between fishers.

The confusion above is related to the MSC system being complex and it being a technical burden. There was also a different type of confusion around the benefits of MSC for each sector. More precisely, the recreational sector of the fishery might have been confused about the need for them to be included in certification. Despite Recfishwest being directly involved in the designing the blueprint for the WA commitment to third party certification, along with WAFIC, many respondents from the recreational sector did not understand what the benefits of certification might be to them (and this confusion apparently remains). Some say, tensions arose between the commercial and recreational sectors because recreational fisheries representatives were not always happy to get involved. However, the opposite was also mentioned; prior tensions between the sectors resolved because they agreed on the environmental standpoint (also due to the shared ‘unit of assessment’ described above). One respondent (#8, fishing association) summarised this: “certification has moved the conversation [between recreational and commercial fishers].” People started talking to each other and the process was giving them the same language and “we didn't end up in the bun-fights that we used to” (Respondent #5, government scientist).

Within the commercial fisheries sector there were some tensions around payment for certification with some respondents indicating there were no (obvious) benefits. Nervousness around the amount of work involved in gaining certification and the fishery’s ability to afford the process without government funding added to the tensions. Some tension was due to concerns about fishery data being released to the public. In addition, tensions were reported about the perceived unsuitability of implementing the MSC standard in local WA fisheries, and that it was unlikely to lead to a positive outcome.

## 4 Discussion

Fisheries management in many countries around the world has improved [1]; however, sustainability issues remain important concerns in many stocks and countries [65]. Globally, market-based incentives such as third-party certification schemes are continuing to recruit fisheries into their schemes. Fisheries that enter certification schemes are required to address any sustainability issues before becoming certified and maintain environmental performance after certification has been achieved. The number of certified fisheries is growing [18] and many different types of certification schemes are now available [29]. Even though debate remains over the effectiveness of market-based mechanisms to enhance environmental management [66–68], there is some empirical evidence of environmental improvements and sustainability outcomes due to certification [28, 61].

The focus and remit of different fisheries certification and rating schemes is increasingly broad. Consideration of other sustainability dimensions such as animal welfare, slavery, and safe and ethical employment have also gained a higher profile [69, 70]. Many of these latter issues are particularly relevant in small-scale fisheries in low income countries [71, 72]. This has led to constantly evolving certification schemes and more specialised schemes focussed around certain issues being developed in parallel. For example, certification schemes such as Fair-Trade USA's (FTUSA) Capture Fisheries Program are aiming to fill some of the gaps in supporting small scale fishery improvements and redistributing ecolabeling benefits [72]. But more broadly, there is a need to frame and analyse these ethical issues and to systematically test and hypothesise solutions for broadening the focus of certification schemes.

Foremost, to continue to verify sustainability outcomes through third party certification, there is a need to understand the impact of certification, beyond the direct, intended outcomes. Gaining insight into the types of impacts that drive (or provide incentives to) stakeholders to become certified and understanding the social, economic, and institutional impacts of certification are key. Our research indicates that in seven Australian MSC certified fisheries captured in this study the most often mentioned driver to become certified is to improve the fisheries sustainability credentials and gain or maintain social licence (or social acceptability) within the fishing community. The economic incentive was the second most important driver, but price premium was not key [73]. These two drivers were relatively more important to the fisheries that had not received government funding to get certified than those who had benefitted from the funding. Because the WA government made funding available for fisheries to become certified the results of our study are very much context specific. However, some general observations can be made especially given there were no significant differences in the responses from individuals from fisheries that received funding to become certified and those that didn’t.

In Western Australia, most fisheries included in this study do not sell their product with the MSC label. The only other way of benefiting of a price premium for being sustainable, without an ecolabel on the product, is if there is widespread awareness in the consumers about the fishery being certified, as well as clear ability to recognise products from that fishery. So, even if there was a premium to be had, it is unlikely that they would receive this without the label, especially on foreign markets, and access to new markets tied to certification is unlikely to occur without a label. Instead, market access and product differentiation were the main economic incentives to participate in certification.

When certification schemes were first implemented globally (in the 1990’s), economic incentives and sustainability outcomes were expected to be the predominant drivers for becoming certified [74] hypothesizing that participant fisheries could capture attractive price premiums. The costs of certification (including ongoing, direct, and indirect costs) were expected to be at least balanced, if not exceeded, by the benefits obtained through price premiums [40]. Early mover fisheries, i.e., the first to sell a particular species on a particular market, indeed have been shown to benefit from retail price premiums [45]. It can be expected that, as more fisheries become certified around the world, it is less likely for a new fishery to capture the first mover advantage, unless it is for a species that is new to the MSC program. Instead, market access and market retention can be expected to become more predominant incentives. Such price premiums can be more easily identified in standard market data streams than other economic benefits, such as improved market access or expanded market share [45]. This may explain why there is high retention of certified fisheries within the MSC program, i.e., fisheries choose to remain certified at the end of the 5 year cycle and undergo re-assessment, including the need for new improvements if the Standard is revised to include new requirements the fishery doesn’t meet.

In WA, the western rock lobster fishery was the global first mover in MSC certification (and one of the case study fisheries in this research). However, the price premium advantage is currently not realised in this fishery. The price for western rock lobster has risen dramatically but this increase is not attributable to MSC certification, even though the fishery did previously capture a price premium when the western rock lobster fishery was selling in Europe. The lack of a certification-related price premium for western rock lobster is largely because the main export market for Australian lobsters does not demand certification [36, 45]. However, some survey respondents in this research alluded to the possibility that Chinese consumers may develop a stronger preference for sustainability. Even though the fishery does not currently sell their product with the MSC label, they have recently recertified for the 4^th^ time at an estimated certification cost of less than 1 cent (AUD) per kilo [75]. The recertification of the western rock lobster fishery suggests that the non-economic benefits (i.e., non-monetary social, institutional, and environmental benefit) of certification outweigh the costs for this fishery. Maintaining sustainability credentials (i.e. retaining the perception of environmental sustainability of the fishery as distinct from social licence) were perceived to be of benefit in this fishery. A corollary to this that some respondents indicated is that the risk posed by the loss of certification (if they decided not to invest in re-certification) would be large. The risk is high because a choice not to recertify might be misinterpreted by the public as a reduction in the sustainability of the fishery–even if this were not the case.

For the WA fisheries that had their certification funded by the WA government, the reason for the lack of price premium is partly the same as that for the western rock lobster—in that they sell to (domestic and export) markets that do not demand certified product. Prices have historically been high or increasing. However, a lack of price premium for this group of government funded MSC fisheries may also be partly attributable to relative appeal of local and global supply chains that do not demand certification. Direct certification costs were not incurred by this group of fisheries, which may perhaps have reduced the drive to seek premium markets. Lastly, these are mostly small-scale fisheries that may have other constraints on their capability to seek premium markets, such as marketing expertise and human resource availability.

As mentioned above, only a small proportion of fish products from WA is sold with the MSC label for several possible reasons. An important reason already alluded to, is that certification of the supply chain can be complicated if there are many points where product changes hands. Even though Chain of Custody certification (as part of the MSC certification process) is less expensive to obtain than the fishery certificate, the most challenging stage of the process seems to be for harvesters to switch to buyers with a certificate or convincing existing buyers to obtain a certificate. Fisheries with vertically integrated supply chains appear to be an enabling factor for using the consumer-facing label because there is more control over the supply chain, and it is easier (and perhaps cheaper) to fully certify a shorter supply chain. Also, in these vertically integrated fisheries, any premiums (or costs) are enjoyed by the consolidated company without using the market to distribute them across multiple supply chain actors. Recognition and appropriate punishment for breaches in the labelling laws [76] or improper use of a certification logo are important risks [77] and legal and scientific investment may help identify options for non-vertically integrated supply chains. However, the only such example in this study is for a fishery that has a strong incentive for ecolabelling as it was notoriously subjected to consumer boycott campaigns due to illegal fishing. Moreover, several fisheries in the MSC program that are not vertically integrated use the ecolabel as well, so it is likely an enabling factor but not the only reason for the difference in economic benefits with the other fisheries in the study.

In contrast to the economic impact (which have largely not eventuated for WA fisheries), social, institutional, and environmental impacts of MSC certification were observed and largely perceived to have been positive. Social acceptability and social licence, both of which were realised in WA, seem to be important drivers to become certified. However, there was also a view that neither the fishery nor the government’s management will really know if they have social licence until it comes under threat or they lose it, and that the ‘jury is still out’ on the issue of social acceptability and social licence.

Nevertheless, the importance of the non-monetary impacts of certification are evident in our research from the perceived relationship between the costs and the benefits of certification. The monetary (economic) benefits of certification only outweigh the costs in certain types of fisheries. Moreover, opinion is divided almost 50:50 on whether the combined non-monetary and monetary benefits outweigh the costs. However, it is important to note that only a small minority indicated that the costs simply outweigh any benefits obtained from certification. These results may be partly attributable to the role of government support in the implementation of MSC certification [78].

This indeterminate result on the costs and benefit ratio for MSC certification may pose a dilemma in attracting new and particularly small fisheries to MSC certification. The barrier of upfront financing to become certified may be lowered (as it was through government funding in WA), but it may not mean that the economic (monetary) benefits will outweigh the ongoing costs after certification is first obtained. This is especially true if a proactive initiative to seek out opportunities of using the ecolabel is lacking or missing. Nevertheless, the non-monetary benefits are perceived to be present and to have been facilitated by the government enabling businesses to join the scheme. Communicating the importance of these potential non-monetary benefits (perhaps by estimation of their monetary value) and potential consideration of other ethical issues may ensure an enduring role for voluntary fisheries certification in creating value for fishery participants.

## Supporting information

S1 File Survey questions. (DOCX)

S1 Table Descriptions of the different drivers for certification. (DOCX)

S2 Table The number of mentions of different themes and whether the impact of MSC certification was perceived as positive, no impact, or negative (n = 33). (DOCX)
