# Correction: Shifting focus: The impacts of sustainable seafood certification

**DOI:** 10.1371/journal.pone.0235602

**Published:** 2020-06-26

**Authors:** Ingrid van Putten, Catherine Longo, Ashleigh Arton, Matt Watson, Christopher M. Anderson, Amber Himes-Cornell, Clara Obregón, Lucy Robinson, Tatiana van Steveninck

In the Author Contributions, Catherine Longo should be listed as one of the persons who contributed to Conceptualization and Funding acquisition.

The following information is missing from the Funding statement: CL received financial support from the Walton Family Foundation.
