# Supplementary material for: Shifting focus: The impacts of sustainable seafood certification
Source: PLoS One. 2020 May 20;15(5):e0233237. doi: 10.1371/journal.pone.0233237 (PMC7239462; doi:10.1371/journal.pone.0233237)
Supplement: S1 File — (DOCX) [file pone.0233237.s001.docx]

# File S1: Survey questions

**Questions about you and your fishery**

1. Which MSC certified fishery are you affiliated with?

___________________________

1. What is your role in this fishery?
   - Individual fisher
   - Fishing association or cooperative representative
   - Government manager
   - Government fisheries scientist
   - Fisheries researcher (in academia)
   - Processor
   - Wholesaler
   - Retailer
   - Exporter
   - Non Government Organisation (NGO)
   - MSC staff
   - Other: __________________________________________
2. How many years have you participated in, or worked with this main certified fishery?

______________________

1. Were you involved in the **pre-assessment** process for this certified fishery?
   - Yes, I was formally involved in the pre-assessment process
   - Yes, I directly participated in the pre-assessment process
   - No, I was not involved in the pre-assessment process
2. For the main certified fishery you identified above were you involved in the full assessment process?
   - Yes, I was formally involved in the full-assessment process
   - Yes, I directly participated in the full-assessment process
   - No, I was not involved in the pre-assessment process
3. Do you participate in any other MSC certified fisheries?
   - I do not participate in any other fisheries
   - I also participate in the following fishery(s):

__________________________

__________________________

__________________________

1. For which of these fisheries did you participation in the pre-assessment process

______________________

1. For which of these fisheries did you participation in the full-assessment process

______________________

1. Please indicate the current status of this fishery (these fisheries) with respect to certification

___________________________________________________________________________________

**The pre-and full assessment process**

1. Why did the fishery seek certification? Please identify the three main reasons.

1 ___________________________________________________________________________________

2 ___________________________________________________________________________________

3 ___________________________________________________________________________________

1. Was there anything happening in the fishery that might have also been a driver for the fishery to get certified? (tick any that apply)

__________________________________________________________________________________

1. Thinking back to the time before the fishery was certified - which stakeholder group(s) started the momentum to get certified? (please tick any that apply)
   - Individual fishermen
   - Fishing cooperative or association
   - Fisheries managers
   - Fisheries scientists
   - Processor
   - Wholesaler
   - Exporter
   - NGO
   - MSC staff
   - Established retail customer
   - New retail customer
   - Other: _________________________________________
2. Which stakeholder groups led the pre-assessment process

____________________________________________________________________________

1. Which stakeholder groups the full assessment process

____________________________________________________________________________

1. Which stakeholder group(s) contributed significant **financial** support to the **pre- assessment** phase of the certification process? *(Tick any that apply).*
   - Individual fishermen
   - Fishing cooperative or association
   - Fisheries managers
   - Fisheries scientists
   - Processors
   - Wholesalers
   - Exporters
   - NGOs or foundations
   - Established retail customers
   - New retail customers
   - Other: _________________________________________
2. Which stakeholder group contributed significant **financial** support to the **full assessment** phase of the certification process? Check all that apply.
   - Individual fishermen
   - Fishing cooperative or association
   - Fisheries managers
   - Fisheries scientists
   - Processors
   - Wholesalers
   - Exporters
   - NGOs or foundations
   - Established retail customers
   - New retail customers
   - Other: ________________________________________

**Agreements and disagreement among participants**

1. Before going through certification, was there any tension or disagreement among **your** stakeholder group whether the fishery should go through the certification process?
   - None
   - A small amount
   - A moderate amount
   - A considerable amount
   - Persistent, ongoing tension or disagreements

a. If you indicated there were conflicts, differences or disagreements before certification, what is the reason for this?

_______________________________________________________________________________

1. Before going through certification, was there (dis)agreement among stakeholder groups (i.e. fishers/ managers/ NGO/ processors etc.) whether the fishery should go through the certification process?
   - Everyone agreed on going through certification
   - Most agreed
   - About half agreed
   - Most disagreed
   - No-one agreed on going through certification
2. What were the principal points of agreement or disagreement about going through the certification process?

____________________________________________________________________________

1. Were there any points of confusion about the pre- and full assessment process?

____________________________________________________________________________

1. Did the assessment process create or resolve any **new** tensions between different stakeholder groups that weren't there before going through certification? (please tick one)
   - No new tension was created
   - Small amount of new tension was created
   - Moderate amount of new tension was created
   - Considerable amount of new tension was created
   - A lot of new tension was created
2. What were the issues that created this new tension?

____________________________________________________________________________

1. Do you think that the views of your stakeholder gro**up** were fully considered during the assessment process?
   - Yes, all would think their views were fully considered
   - Yes, the majority would think their views were fully considered
   - About half would think their views were fully considered
   - No, a minority would think their views were fully considered
   - No, none would think their views were fully considered
2. If no, can you explain why you think their views were not adequately considered?

___________________________________________________________________________________

1. Now that this fishery is certified, how satisfied are you with the process of going through the assessment? (please tick one)
   - Very satisfied
   - Satisfied
   - Neither satisfied nor dissatisfied
   - Dissatisfied
   - Very dissatisfied

**Anticipated and realized effects of certification**

1. Which were the top three benefits (starting with the most important) that you were expecting to get from certification?

1 Benefit __________________________________________________________________________

2 Benefit __________________________________________________________________________

3 Benefit __________________________________________________________________________

1. Can you please indicate if these expected benefits were also realized?

Benefit 1 was realized/not realized _____________________________

Benefit 2 was realized/not realized _____________________________

Benefit 3 was realized/not realized _____________________________

1. Please indicate if which way the **economic** and **supply chain** effects of MSC certification have been realized or not (tick one for each row). The effects are all phrased in the positive – but the opposite answer can be true

|  | This was expected | | | This was **not** expected | | |  |
| --- | --- | --- | --- | --- | --- | --- | --- |
| **Economic and/or supply chain effect** | has remained unchanged | has improved | has worsened | has remained unchanged | has improved | has worsened | I don’t know |
| *Higher* price paid to fishers |  |  |  |  |  |  |  |
| *Higher* price paid to processors / exporters |  |  |  |  |  |  |  |
| *Increased* demand for fish |  |  |  |  |  |  |  |
| *Competitive* advantage over uncertified fisheries |  |  |  |  |  |  |  |
| *Greater* market access |  |  |  |  |  |  |  |
| *Maintenance* of market access |  |  |  |  |  |  |  |
| *More* niche or value added products |  |  |  |  |  |  |  |
| *Greater* access to credit (with banks) |  |  |  |  |  |  |  |
| *Better* product traceability |  |  |  |  |  |  |  |
| *Greater* awareness of MSC in Australia |  |  |  |  |  |  |  |
| Supermarket *recognition* of MSC brand |  |  |  |  |  |  |  |
| *Promotion* of the fishery by MSC |  |  |  |  |  |  |  |
| *Improvement* in the distribution of benefits over supply chain |  |  |  |  |  |  |  |
| Other (please list) |  |  |  |  |  |  |  |

1. Please indicate if which way the **social** effects of MSC certification have been realized or not (tick one for each row)

|  | This was expected | | | This was **not** expected | | |  |
| --- | --- | --- | --- | --- | --- | --- | --- |
| **Social effect** | has remained unchanged | has improved | has worsened | has remained unchanged | has improved | has worsened | I don’t know |
| *Improved* understanding of the fishery |  |  |  |  |  |  |  |
| *More* employment in the fishery |  |  |  |  |  |  |  |
| *More positive* interactions between stakeholders |  |  |  |  |  |  |  |
| *Improved* reputation in the community |  |  |  |  |  |  |  |
| *More* trust in the fishery by the community |  |  |  |  |  |  |  |
| *Greater* credibility of the industry |  |  |  |  |  |  |  |
| *Reduced* criticism of the fishery (especially by NGOs) |  |  |  |  |  |  |  |
| *Improved* recreational and commercial fisheries interaction |  |  |  |  |  |  |  |
| *Improved* dialogue between stakeholder groups |  |  |  |  |  |  |  |
| *More* pride in participating in the fishing sector |  |  |  |  |  |  |  |
| *Decreased* exclusivity of access |  |  |  |  |  |  |  |
| *Reduced* interaction with species of cultural / recreational value |  |  |  |  |  |  |  |
| Other |  |  |  |  |  |  |  |

1. Please indicate if which way the **environmental** effects of MSC certification have been realized or not (tick one for each row)

|  | This was expected | | | This was **not** expected | | |  |
| --- | --- | --- | --- | --- | --- | --- | --- |
| **Environmental effect** | has remained unchanged | has improved | has worsened | has remained unchanged | has improved | has worsened | I don’t know |
| *Increased* harvest levels (catches) |  |  |  |  |  |  |  |
| *Greater* fishery participant knowledge of sustainable practices |  |  |  |  |  |  |  |
| *Improved* practices with respect to threatened and endangered species |  |  |  |  |  |  |  |
| *Improved* fishery environmental footprint |  |  |  |  |  |  |  |
| A greater number of environmental objectives achieved |  |  |  |  |  |  |  |
| Other |  |  |  |  |  |  |  |

1. Please indicate if which way the **management and political** effects of MSC certification have been realized or not (tick one for each row)

|  | This was expected | | | This was **not** expected | | |  |
| --- | --- | --- | --- | --- | --- | --- | --- |
| **Management and political** **effect** | has remained unchanged | has improved | has worsened | has remained unchanged | has improved | has worsened | I don’t know |
| *Greater* influence in the political or regulatory process |  |  |  |  |  |  |  |
| *Better* governance of the industry |  |  |  |  |  |  |  |
| *Improved* management practices |  |  |  |  |  |  |  |
| *Greater* government confidence in the industry |  |  |  |  |  |  |  |
| *Better* recognition of sustainable practices |  |  |  |  |  |  |  |
| *More* streamlined (efficient) assessment processes |  |  |  |  |  |  |  |
| *More* transparent management processes |  |  |  |  |  |  |  |
| *Recognition* of improved practices that were already implemented before MSC |  |  |  |  |  |  |  |
| Other |  |  |  |  |  |  |  |

1. Were there any other **unexpected consequences** from MSC certification (these can be positive or negative)?

___________________________________________________________________________________

1. Do you believe that for your fishery, the benefits of certification outweigh the costs of becoming certified (including monetary and non-monetary costs of certification assessment and costs of coming into compliance with the certification conditions)?

- Yes, the benefits of certification are much greater than the costs.
- Yes, the benefits of certification are slightly greater than the costs.
- The benefits of certification are even with the costs of becoming certified.
- No, the costs are slightly greater than the benefits.
- No, the costs are much greater than the benefits.

1. Have any of the following developments happened in your fishery and how do you think those events relate to MSC certification?

|  | This has not happened | This has happened and is **not** linked to MSC | This has happened and is linked to MSC | I don’t know |
| --- | --- | --- | --- | --- |
| Marine heatwave affecting stocks |  |  |  |  |
| Extreme environmental events |  |  |  |  |
| Reduced operating cost |  |  |  |  |
| Extended fishing season |  |  |  |  |
| Less time spent at sea |  |  |  |  |
| Higher profits |  |  |  |  |
| More fishing to market |  |  |  |  |
| More product types produced |  |  |  |  |

# Relationships within the fishery

1. What best characterizes your stakeholder group's current working relationship with the following other actors? (Please tick each row)

|  | The working relationship has not changed | I have a better working relationship | I have a worse working relationship | I have no relationship with them | I don’t know |
| --- | --- | --- | --- | --- | --- |
| Certified fishers | ❑ | ❑ | ❑ | ❑ | ❑ |
| Uncertified fishers | ❑ | ❑ | ❑ | ❑ | ❑ |
| Wholesale buyers | ❑ | ❑ | ❑ | ❑ | ❑ |
| Retail buyers | ❑ | ❑ | ❑ | ❑ | ❑ |
| NGOs | ❑ | ❑ | ❑ | ❑ | ❑ |
| Fisheries managers | ❑ | ❑ | ❑ | ❑ | ❑ |
| Fishery scientists | ❑ | ❑ | ❑ | ❑ | ❑ |
| MSC managers | ❑ | ❑ | ❑ | ❑ | ❑ |
| MSC scientists | ❑ | ❑ | ❑ | ❑ | ❑ |
| Other non-fisheries stakeholders (community) | ❑ | ❑ | ❑ | ❑ | ❑ |

1. What aspect of certification led to **improved** working relationships being created?

___________________________________________________________________________________

1. What aspect of certification led to the **worsening** of working relationships?

___________________________________________________________________________________

1. How would you best describe your interactions with the local MSC representative?
   - I have not had direct interactions with MSC staff
   - Extremely positive
   - Positive
   - Neutral
   - Negative
   - Extremely negative
2. How would you best describe your interactions with the MSC staff in London?
   - I have not had direct interactions with MSC staff
   - Extremely positive
   - Positive
   - Neutral
   - Negative
   - Extremely negative
3. How would you best describe your interactions with the certification bodies (CABs)?
   - I have not had direct interactions with MSC staff
   - Extremely positive
   - Positive
   - Neutral
   - Negative
   - Extremely negative
4. Any other comments

__________________________________________________________________________________________
