# Supplementary material for: Shifting focus: The impacts of sustainable seafood certification
Source: PLoS One. 2020 May 20;15(5):e0233237. doi: 10.1371/journal.pone.0233237 (PMC7239462; doi:10.1371/journal.pone.0233237)
Supplement: S1 Table — (DOCX) [file pone.0233237.s002.docx]

# Table S1: Descriptions of the different drivers for certification

| **Drivers for certification** | Description | **Mentions (number)** |
| --- | --- | --- |
| **Sustainability credentials and SLO** | Gaining social licence for fishery activities and being world leaders in terms of sustainability credentials | **27** |
| **Economic incentive** | economic advantage from certification that included differentiation, market access, marketing advantages and price premiums | **17** |
| **Funding availability** | availability of government funding to get certified |  |
| **Improve management outcomes** | Improving management through changing environmental impact and improving environmental outcomes | **11** |
| **Outside pressure** | international and social pressure to improve management | **6** |
