# Supplementary material for: Shifting focus: The impacts of sustainable seafood certification
Source: PLoS One. 2020 May 20;15(5):e0233237. doi: 10.1371/journal.pone.0233237 (PMC7239462; doi:10.1371/journal.pone.0233237)
Supplement: S2 Table — (DOCX) [file pone.0233237.s003.docx]

# Table S2: The number of mentions of different themes and whether the impact of MSC certification was perceived as positive, no impact, or negative (n= 33).

| **Impact sub themes** | **positive impact** | **no impact** | **negative impact** |
| --- | --- | --- | --- |
| **Economic** | **35** | **65** | **6** |
| brand recognition | 2 | 20 |  |
| business costs | 1 |  | 3 |
| chain of custody | 1 | 7 | 3 |
| credit access | 3 | 1 |  |
| differentiation | 2 | 2 |  |
| economic advantage | 1 | 7 |  |
| employment |  | 1 |  |
| market access | 7 | 9 |  |
| market advantage | 1 | 3 |  |
| marketing | 9 | 3 |  |
| price premium | 5 | 10 |  |
| product value | 2 | 2 |  |
| trust | 1 |  |  |
| **Environment** | **35** | **6** | **3** |
| credibility | 1 |  | 1 |
| data availability | 2 |  |  |
| environmental impact | 14 |  | 2 |
| environmental management | 7 | 1 |  |
| environmental performance | 2 | 4 |  |
| environmental quality | 2 |  |  |
| harvest strategy | 2 | 1 |  |
| knowledge level | 3 |  |  |
| research opportunities | 2 |  |  |
| **Institutional** | **66** | **11** | **8** |
| brand recognition |  | 1 |  |
| communication | 3 |  |  |
| community support | 2 |  |  |
| data availability | 2 |  | 1 |
| engagement | 1 |  |  |
| environmental impact | 1 | 1 |  |
| environmental management | 4 | 1 |  |
| environmental outcomes | 3 | 1 |  |
| funding (resource) availability | 8 |  | 1 |
| harvest strategy | 5 |  |  |
| management impact | 4 | 1 |  |
| management practices | 3 | 1 |  |
| management process | 4 | 1 | 3 |
| negotiation | 2 |  |  |
| political influence | 7 | 3 | 1 |
| political support | 3 |  |  |
| transparency | 14 | 1 | 2 |
| **Social** | **96** | **25** | **4** |
| communication | 15 | 2 |  |
| community support | 4 |  |  |
| credibility | 3 |  |  |
| data availability | 1 |  |  |
| employment | 1 |  |  |
| engagement | 5 |  |  |
| industry image | 3 | 1 |  |
| knowledge level | 16 | 3 |  |
| marketing | 1 | 1 |  |
| pride | 7 | 4 |  |
| research opportunities | 1 | 1 | 1 |
| social impact | 1 | 2 | 1 |
| social licence | 23 | 7 |  |
| social responsibility | 1 |  |  |
| stakeholder collaboration | 12 | 2 | 2 |
| trust | 2 | 2 |  |
| **Grand Total** | **232** | **107** | **21** |

# 
